# Comparison of Coagulation Parameters in Arterial and Venous Blood in Cardiac Surgery Measured Using the Quantra System

**DOI:** 10.1053/j.jvca.2018.08.201

**Published:** 2018-08-31

**Authors:** Danja S. Groves, Deborah A. Winegar, Lucas G. Fernandez, Julie L. Huffmyer, Francesco Viola

**Affiliations:** * Department of Anesthesiology, University of Virginia Health System, Charlottesville, VA; † HemoSonics, LLC, Charlottesville, VA

**Keywords:** arterial blood, venous blood, cardiac surgery, coagulation testing, Quantra System

## Abstract

**Objective:**

Perioperative coagulation testing often is performed with arterial samples even though device reference ranges typically are established in venous samples. Although limited studies exist comparing coagulation parameters across sampling sites, viscoelastic testing devices have demonstrated some differences. The objective of this study was to compare coagulation parameters determined using the Quantra System for venous and arterial samples.

**Design:**

Prospective, observational study.

**Setting:**

Tertiary care university hospital.

**Participants:**

The study comprised 30 adult patients undergoing cardiac surgery.

**Interventions:**

Paired arterial and venous samples were obtained at 2 of the following time points: baseline, during bypass, or after protamine reversal of heparin. Quantra measurements included Clot Time (CT), Heparinase Clot Time (CTH), Clot Time Ratio (CTR), Clot Stiffness (CS), and Fibrinogen (FCS) and Platelet (PCS) Contributions to clot stiffness.

**Measurements and Main Results:**

The relationship and agreement between matched data pairs were established and statistical analysis was performed via paired *t* tests. CTR, CS, FCS, and PCS were unaffected by the sampling site, whereas CT and CTH demonstrated statistically significant differences between arterial and venous samples (p < 0.001). Arterial clot times were prolonged relative to the venous ones with a mean percent error of 14.2 % and 11.9 %, respectively. These results are in general agreement with those reported for other viscoelastic testing devices.

**Conclusions:**

This study demonstrates that Quantra clot stiffness-based parameters (CS, FCS, PCS) are unaffected by sampling site, whereas the clot time parameters (CT and CTH) show good correlation in the presence of a bias. CTR, a ratio of CT and CTH, also is unaffected.

COAGULATION TESTING often is performed in cardiac surgery and other major surgical procedures to guide anticoagulant therapies or to aid in the management of perioperative coagulopathies.^1–4^ It is well established that accurate and timely management of coagulation function in these patients results in improved outcomes. A constellation of devices and assays currently are available to support the clinical need, which include well-established coagulation assays, such as active partial thromboplastin time (aPTT), prothrombin time/ international normalized ratio (PT/INR), activated clotting time (ACT), and viscoelastic testing devices (VETs), such as TEG 5000 (Haemonetics Corporation, Braintree, MA) and ROTEM delta (Tem Innovation GmbH, Munich, Germany).^1,3^

The Quantra Hemostasis Analyzer (Quantra) (HemoSonics LLC, Charlottesville, VA) is a novel point-of-care device that measures changes in the viscoelastic properties of a whole blood sample during coagulation.^5^ These changes are representative of the complex functional interactions between the blood’s plasma factors and the cellular components. The Quantra’s performance recently was tested and demonstrated in a multicenter study involving patients undergoing cardiac and major spine reconstruction surgeries.^6–8^

Reference ranges for these diagnostic devices are established to determine the specific performance characteristics in a normal, healthy population and are used to define clinically relevant thresholds that can be used to inform appropriate medical treatment. However, even though reference ranges typically are established using venous blood samples, perioperative testing can be performed with either arterial or venous blood samples, depending on the specific surgical procedure and available sampling access point(s). To this date, despite the widespread adoption of these assays, only limited studies have been published to characterize the effects of sampling site on coagulation testing.

An early study on the effects of sampling site in plasma-based coagulation assays indicated some arterial and venous differences in healthy patients but less marked differences in patients with coronary artery disease.^9^ A more recent study found great variability in arterial versus venous activated clotting time measurements in cardiac surgery patients, especially during heparin administration.^10^

With respect to the VET devices, one study reported shorter clot times and stiffer clots in arterial samples compared with venous samples in cardiac surgery patients monitored with the TEG 5000.^11^ This study also found that the arterial and venous TEG parameters are only moderately correlated. Similarly, a study in 30 anesthetized swine demonstrated sampling site differences in most TEG 5000 parameters.^12^ Another study showed little to no differences in the ROTEM delta parameters between arterial and venous samples obtained from orthopedic surgery patients; arterial and venous variables showed strong to very strong correlation except for CT, which showed only moderate correlation between the 2 sample types.^13^ Recently, a study from Tuovila et al. with patients undergoing a broad range of surgical procedures reported differences of up to 19% for clot time and up to 5.5 % for maximum clot amplitude on paired sets of arterial and venous samples on the TEG 5000.^14^

In this article, the authors report the results of a prospective, observational pilot study aimed at assessing whether differences exist between Quantra System measurements obtained in arterial and venous whole blood samples. The study was performed at the University of Virginia Medical Center (Charlottesville, VA) and involved adult patients undergoing elective cardiac surgery using cardiopulmonary bypass (CPB).

## Methods

The protocol for this prospective, observational study was approved by the University of Virginia Institutional Review Board for Health Sciences Research (protocol approval number HSR19008) and complied with the Declaration of Helsinki. The study was registered under clinical trial number NCT02978872. Written, informed consent was obtained from all enrolled patients.

Intraoperative and postoperative care was at the discretion of the anesthesiology and surgical teams and was not influenced by this study. Results from the Quantra System were blinded and not available to the clinical team. Clinical decisions regarding patient care and transfusion of autologous and allogeneic blood products were based on established institutional practice guidelines.

### Study Population

Thirty patients older than 18 years who were scheduled for elective cardiac surgery with CPB between August 2016 and February 2017 were enrolled in the study. Surgical procedures included coronary artery bypass grafting surgery, valve replacement surgery, aortic surgery, and ventricular assist device placement or replacement. Preoperative use of anticoagulants or antiplatelet drugs did not preclude patient enrollment. Patient demographic data and surgical details are summarized in Table 1.

Although this was a pilot study aimed at providing initial performance characteristics, the sample size calculation was based on the Quantra measurement of CT. As discussed further, a CT measurement is not available during bypass, therefore it represents the parameter with the fewest paired measurements. With the assumption of a paired mean difference of 8.4 seconds (slightly greater than the estimated precision of CT), the minimum sample requirement was 40 paired arterial and venous measurements with an alpha level equal to 0.05 and 80 % power. Note that the number of patients was based on the size of similar studies reported for other VET devices.

### Blood Sampling

For each enrolled patient, matched pairs of arterial and venous samples were drawn at baseline (preoperatively, after the induction of anesthesia and before incision) and at 1 of the following 2 potential time points: (1) before the end of bypass and while the patient was still heparinized or (2) 10 minutes after protamine reversal of heparin. Roughly, an equal number of samples was obtained for the bypass and postbypass time points.

Whole blood samples were drawn into 2.7 mL citrated tubes from existing arterial catheters (20 G radial artery catheter; Teleflex, Wayne, PA) or central venous catheters (VIP multilumen Swan Ganz catheter; Edwards Lifesciences, Irvine, CA). All the catheters from which blood samples were drawn were not coated with anticoagulant, nor was heparin used in the flush system. In order to avoid contamination with the flush system, all venous samples were obtained after the withdrawal of 10 mL of blood, corresponding to roughlythree times the dead space volume of the catheter. The arterial and venous samples were drawn nearly simultaneously but run in series in the same Quantra System.

### Quantra Testing

The Quantra System used in this study is a next generation point-of-care VET device consisting of an instrument (the Quantra), a single-use disposable cartridge, and external quality control materials. The Quantra measures changes in clot stiffness during coagulation using ultrasound generation and detection of resonance. A detailed description of the deviceand its principles of operation are described elsewhere. ^5,15^

**Fig. 1.**
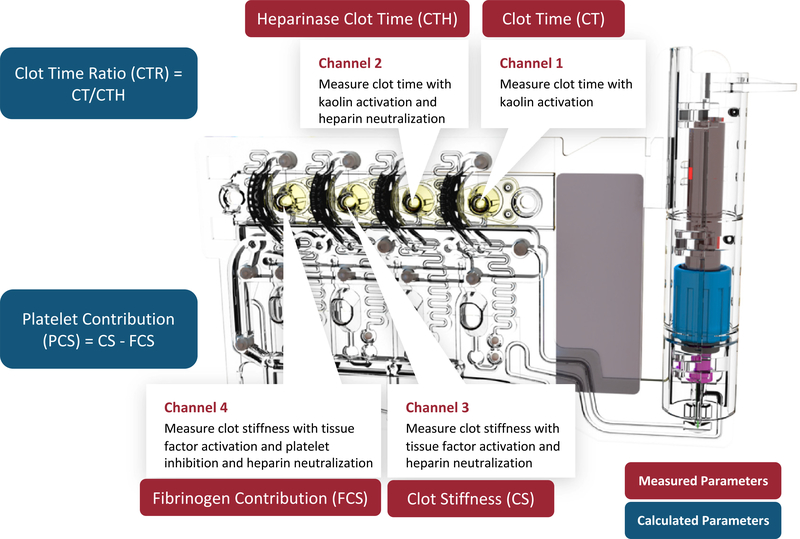
Quantra QPlus Cartridge used in this study. The cartridge consists of four parallel channels with different sets of reagents that are optimized to measure clot time or clot stiffness values. Four parameters are directly measured from the four channels of the cartridge, and two parameters are calculated from the measured ones.

In the present study, the Quantra was used in combination with the QPlus Cartridge (formerly the Surgical Cartridge). The QPlus Cartridge, shown schematically in Fig. 1, contains different combinations of lyophilized reagents in 4 channels that enable 4 independent tests to be run in parallel.^5^Clot times and clot stiffness values obtained from these channelsX are combined to form 6 parameters that depict the patient’s coagulation status; these parameters are summarized in Table 2 along with the respective reference intervals. The parameters directly measured include Clot Time (CT), Heparinase Clot Time (CTH), Clot Stiffness (CS), and Fibrinogen Contribution to clot stiffness (FCS). Clot Time Ratio (CTR) and Platelet Contribution to clot stiffness (PCS) are calculated from the measured parameters. Note that CTH, CS, FCS, and PCS are not affected by the presence of heparin in the sample because the reagents used to obtain these parameters include a heparin neutralizer (heparinase I for CTH and hexadimethrine bromide for CS, FCS, and PCS).

Research use only versions of the Quantra and the QPlus Cartridge were used for the present study. The system was located in close proximity to the patient and operated by the anesthesia staff according to the manufacturer-recommended guidelines. Quality controls provided by the manufacturer were run daily to verify the performance of the analyzer. The surgical team was not informed about the results of the Quantra System and performed the surgical procedures following the institution’s guidelines.

### Statistical Analysis

Data were processed and analyzed using MATLAB, Version 9.2.0, with the Statistics and Machine Learning Toolbox, Version 11.1 (MathWorks Inc., Natick, MA), and SAS, Version 9.4 (SAS Institute Inc., Cary, NC). Differences in the mean values of arterial and venous samples were assessed using a Student t test, with p < 0.05 indicating statistical significance. Bland-Altman plots with 95% confidence intervals also were generated to evaluate the agreement between the 2 sample types. Furthermore, the degree of association between arterial and venous measurements was assessed via standard linear regression analysis. The strength of the correlation (Pearson *r* value) was assessed according to the following definitions: 0.00 to 0.19 (very weak), 0.20 to 0.39 (weak), 0.40 to 0.59 (moderate), 0.60 to 0.79 (strong), and 0.80 to 1.00 (very strong).

## Results

From the 30 enrolled patients undergoing cardiac surgery using CPB, a total of 60 paired arterial and venous blood samples were drawn for comparison. One sample was taken from all patients at baseline. A second paired sample was taken from 16 patients during CPB and from 14 patients post-protamine administration. Analysis of these samples on the Quantra with the QPlus Cartridge yielded 40 pairs of matched arterial/venous CT measurements, 56 CTH pairs, 55 CS pairs, 53 FCS pairs, 52 PCS pairs, and 40 CTR pairs. Three samples (2 venous, 1 arterial) failed to generate results owing to cartridge fill errors and the results from 1 cartridge (0.8%) were eliminated because of reported sample hemolysis. In these cases, the corresponding matched samples were not included for further data analysis. Also, as previously mentioned, the number of CT measurements is lower than other parameters because CT is not reported by the Quantra for blood samples obtained during bypass (ie, in the presence of high heparin concentration).

**Fig. 2.**
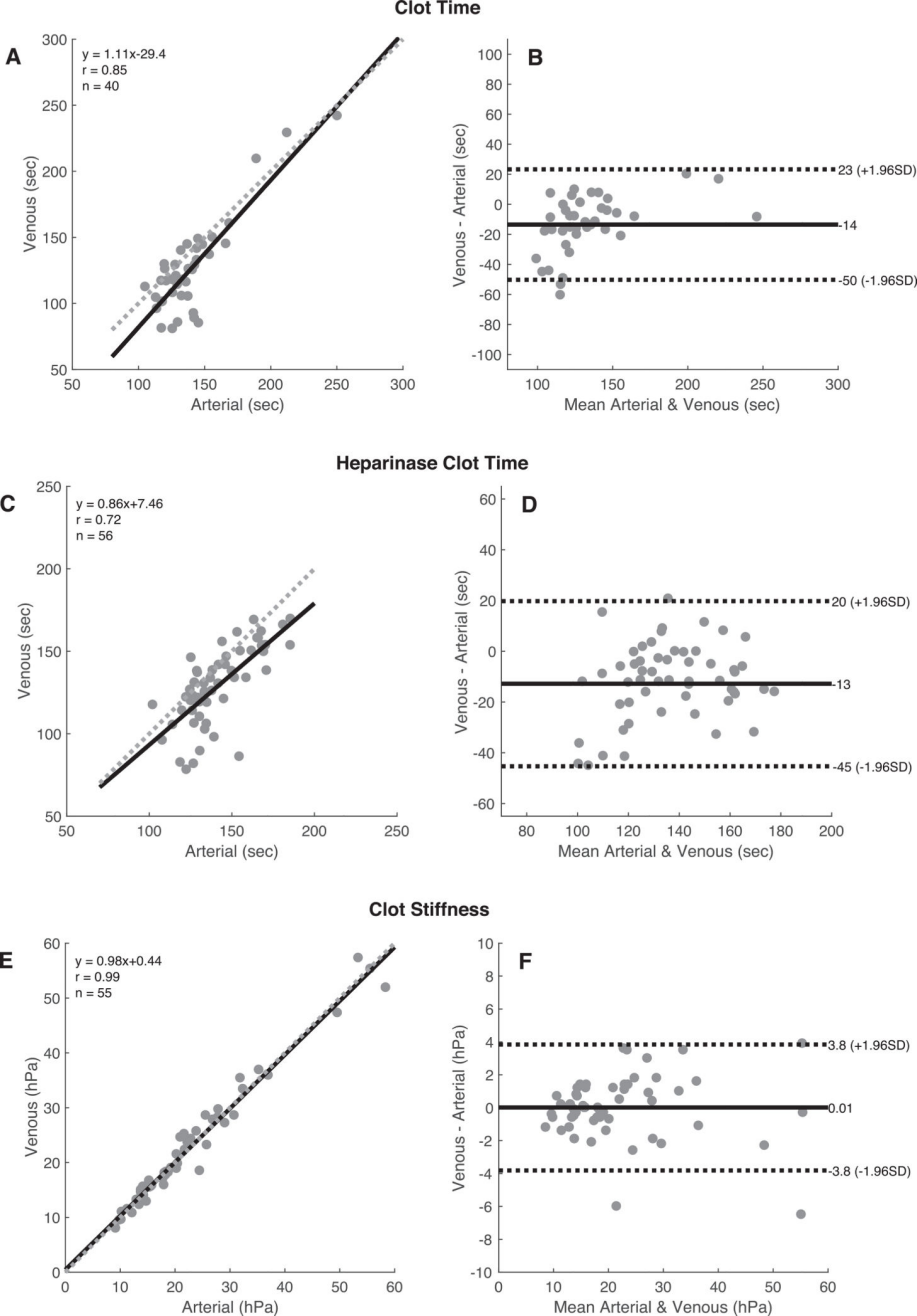

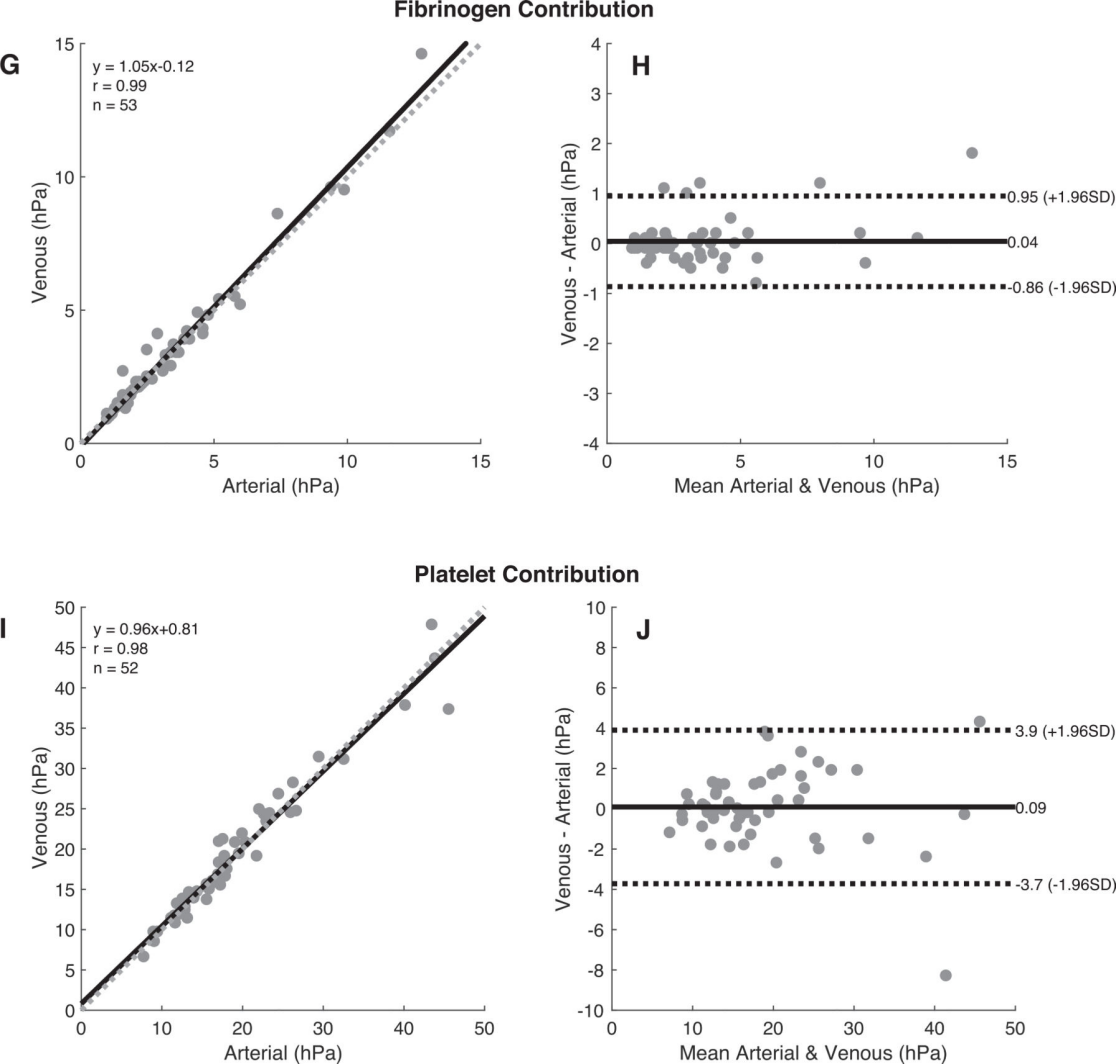
CorrelationandBland-AltmanplotsforthefollowingQuantraQPlusparameters: Clot Time(A and B), Heparinase Clot Time(C and D), Clot Stiffness (E and F), Fibrinogen Contribution to clot stiffness (G and H), and Platelet Contributions to clot stiffness (I and J). The correlation plots include the best linear fit line (*solid line*) and the unitary line (*dotted line*).

Fig. 2 presents a series of plots that show the relationship and linear agreement between matched arterial and venous measurements, including correlation scatter plots and Bland-Altman agreement plots for CT, CTH, CS, FCS, and PCS. These data indicate that there was a very strong and strong correlation between arterial and venous measurements for CT and CTH (*r* = 0.85 and 0.72, respectively). However, for both parameters, the arterial sample typically showed a prolonged clot time compared with the matched venous sample. The observed mean differences were 13.5 seconds and 12.8 seconds for CT and CTH, respectively.

The QPlus clot stiffness-based parameters (CS, FCS, and PCS) showed very strong correlation between the two sample types with *r* values > 0.98 in all cases. In addition, as indicated by the Bland-Altman plots for these parameters, there were no significant measurement biases between the arterial and venous samples.

**Fig. 3.**
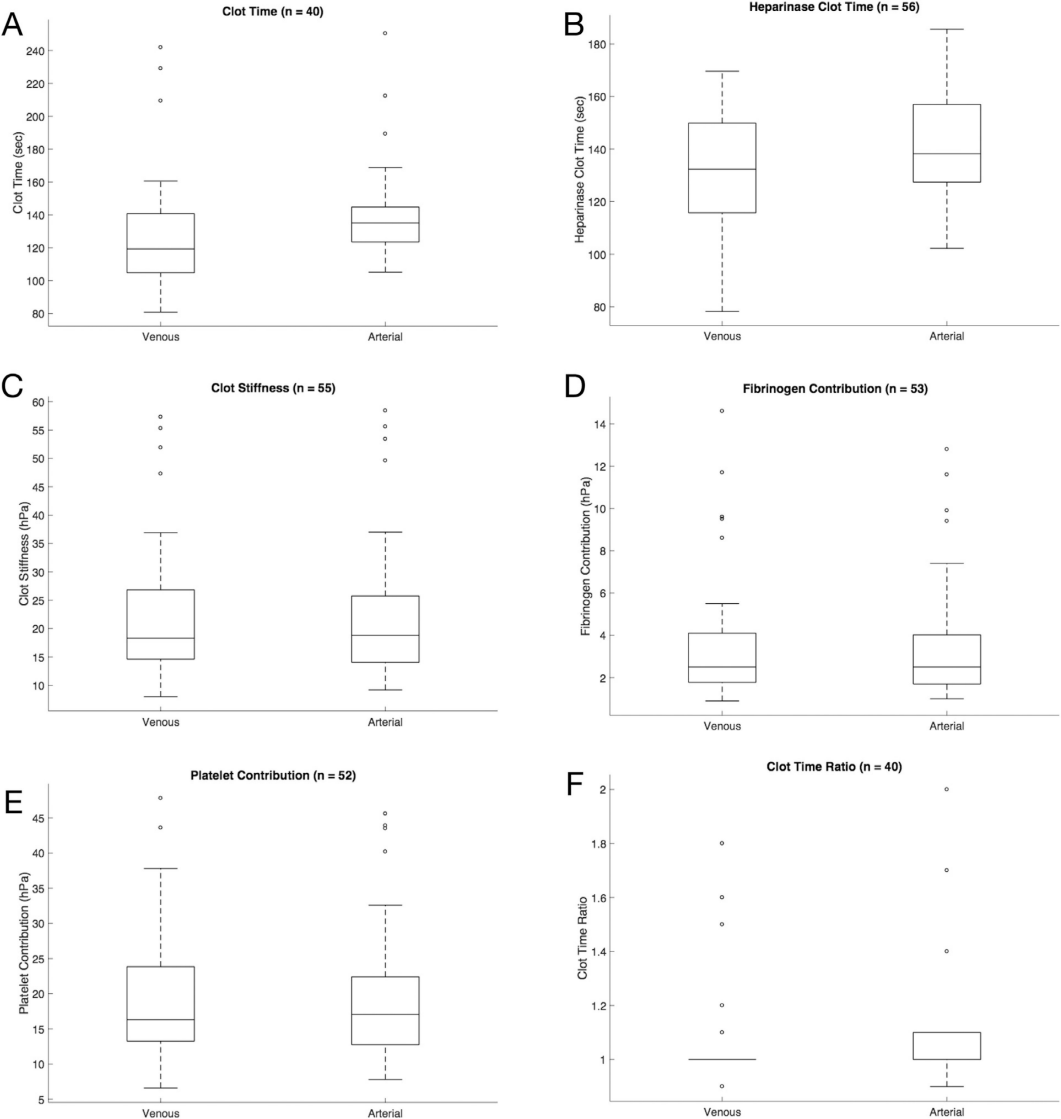
Box-and-whisker plots of the matching venous and arterial samples for the measured and calculated QPlus parameters. In these plots, the upper and lower limits of the boxes represent the interquartile ranges of the data, the line within each box represents the median, and the whiskers extend to data points that are within 1.5× the interquartile range. Outliers are marked with an *open circle*.

Fig. 3 displays the distributions of the arterial and venous parameters as box-and-whisker plots. As expected, the matched CT and CTH parameters showed the presence of a modest time bias between the matched sample pairs (13.5 seconds for CT and 12.8 seconds for CTH). Panels C through E show the matched CS, FCS, and PCS, respectively.

Paired *t* tests were performed on the mean difference values of the matched arterial and venous samples. Data are summarized in Table 3. In agreement with the results previously presented, CS, FCS, and PCS were similar between arterial and venous samples and did not show a statistically significant difference. However, CT and CTH demonstrated a statistically significant difference (p < 0.001). As demonstrated in the Bland-Altman plots, arterial CTs were prolonged relative to the venous ones, with an average bias of 13.5 seconds and 12.8 seconds, respectively.

## Discussion

The relationship and overall agreement between arterial and venous blood with respect to coagulation function is poorly characterized. In critical care settings, for example, either sampling site could be used for patient testing, even though reference ranges and analytical performance, both of which are used to guide clinical interventions, typically are established with venous samples. Herein, the authors describe a pilot observational study aimed at assessing potential differences between the viscoelastic properties of arterial and venous whole blood samples during coagulation as measured using the Quantra System. The Quantra is a novel point-of-care VET system designed to rapidly characterize the functional role of the main coagulation components from a sample of citrated (3.2%) whole blood.

The study was not designed to assess the clinical effect of any potential difference in Quantra parameters determined in arterial versus venous whole blood samples but simply to characterize the magnitude of potential measurement differences. The study involved adult patients undergoing a variety of cardiac procedures with CPB. No venipunctures were used, but samples were obtained from existing venous and arterial catheters placed as part of the patient’s standard of care. Samples were run on the Quantra Hemostasis Analyzer with activation provided by the reagents included in the QPlus Cartridge (see Table 2).

The data presented in Figs. 2 and 3 indicate that although there was strong correlation between the results obtained from arterial and venous samples for CT and CTH, these parameters do not show good agreement with respect to sample site, as demonstrated in the Bland Altman plots. The 95% limits of agreement for CT showed differences of up to 50 seconds, whereas the 95% limits of agreement for CTH showed differences up to 45 seconds. The magnitude of the differences typically was greater at lower values of the venous-derived clot times. The mean percentage error between the matched sample was 14.2% (median 9.1%) for CT and 11.9% (median 8.7%) for CTH. These differences are statistically significant, as demonstrated in Table 3. These results are in general agreement with those reported by other investigators using VET devices in surgical patients. Manspeizer et al.^11^ found statistically significant differences in most output parameters of the TEG 5000 with celite activation in a study involving cardiac patients. The correlation between arterial and venous R times (reaction times or clot times) was only moderate, with an *r* value of 0.44. Tuovila et al.^14^ indicated a mean percent error of 17.1% (median of 9.1%) for kaolin activated R time with the TEG 5000. Also in this case, venous R times were shorter than arterial R times. Oswald et al.^13^ also found a statistically significant difference for the ROTEM delta’s INTEM CT (assay activated via ellagic acid) with moderate correlation between arterial and venous samples (*r* value of 0.519).

CTR is a unique QPlus parameter derived by dividing CT by CTH; CTR was designed to indicate the influence of residual heparin anticoagulation in the patient samples. Although the scatter plot and Bland-Altman plot are not provided for this parameter, it showed good agreement and good relationship with respect to sample type, thus suggesting that the measurement biases present in CT and CTH cancel out each other. The mean percentage error between the matched samples was 0.36% with a median value of 0.03%.

The QPlus parameters based on measurements of clot stiffness6_X8DX_ (ie, CS, FCS, and the derived PCS) exhibited good agreement and very strong correlation between venous and arterial sampling sites (see Fig. 2). The Bland-Altman plots for CS indicate minimal measurement biases with a trend toward larger differences with increasing mean values. The mean percentage errors between the matched sample for CS, FCS, and PCS were 0.63%, 0.81%, and 0.02%, respectively. Previous studies have reported conflicting results with respect to stiffness-based parameters of the TEG and ROTEM systems, with some investigators showing good agreement and linear correlation,^13,14^ whereas others have found statistically significant differences.^11,16^

The mechanisms for the differences observed in the present study and those previously reported in other studies currently are unknown. It has been hypothesized that differences in oxygen content could be a potential source of discrepancy because oxygenated blood is more viscous.^17^ However, studies from Frumento et al. in a cohort of cardiac patients indicated that oxygen content alone cannot account for the magnitude of the reported differences in CT and CS measurements.^16^ Instead this study suggested that shear stress applied to the sample during collection through the catheters can generate platelet activation, resulting in faster clot times and higher clot stiffness values in the venous samples compared with the arterial ones.

The total assay precision (coefficient of variation) observed for the Quantra measurements of CT and CTH determined in whole blood samples collected using venipuncture is in the range of 4% to 9%. This assay variability could account for a portion of the observed measurement biases for the CT and CTH parameters. Although there was no sample randomization in the current study and the arterial sample always was run first, the authors hypothesize that the variation in storage time of the samples was not likely to have contributed to the observed measurement biases. There was an average delay between arterial and venous runs of 22.7 minutes (standard deviation of 7.3 min) with maximum and minimum delays of 52 minutes and 17 minutes, respectively. These delays were not correlated with the observed arterial/venous differences in CT and CTH. Finally, and most likely, given the variable inner diameters of the catheters used to collect samples in this study, it is possible that the generation of high shear stress at the time of collection could have affected coagulation function before testing was initiated. Potential differences in residual heparin or residual catheter “flush” in the test tubes was not considered a viable mechanism for the observed variability because those effects were accounted for in the study design with a 10 mL draw from the catheters and by the assay design with heparin neutralization.

## Conclusions

The results of this study indicate that there were no appreciable differences with respect to sampling site (venous or arterial) in the following QPlus Cartridge parameters using the Quantra Hemostasis Analyzer: CTR, CS, FCS, and PCS. The FCS and PCS parameters are designed to identify deficiencies in fibrinogen and platelet functions that might led to excessive bleeding. The CTR parameter is designed to indicate the presence of residual heparin. The QPlus parameters based on measurements of clot times do exhibit some arterial/venous differences. Additional studies are required for further investigations of this phenomenon.

## Figures and Tables

**Table 1:** Study Demographics

Characteristic	Value (Mean ± Standard Deviation/Number of Patients [%])
Male sex (n)	22/30 (73.3)
Age (y)	62.2 ± 11.5
Weight (kg)	88.9 ± 18.9
Procedure (n)	
CABG	10 (33.3)
Valve repair or replacement	10 (33.3)
CABG/valve repair or replacement	3 (10.0)
VAD	3 (10.0)
Other	4 (13.3)
CPB time (min)	124.1 ± 60.1
Cross-clamp time (min)	76.0 ± 43.7
Preoperative medications (n)	
Aspirin	20 (66.6)
Heparin	14 (46.6)
V-K antagonists	6 (20)
P2Y12 antagonists	2 (6.6)
Direct thrombin inhibitors	0 (0)
FXa inhibitors	1 (0.3)
Volume of cell saver administered (mL)	1095.2 ± 921.8

Abbreviations: CAGB, coronary artery bypass grafting; CPB, cardiopulmonary bypass; FXa, Factor Xa; VAD, ventricular assist device; V-K, vitamin K.

**Table 2:** Parameters Output by the Quantra and QPlus Cartridge

QPlus Parameter	Units	Description	Reference Range
Clot time (CT)	Seconds	CT measured with kaolin activation	104–166 s
Heparinase clot time (CTH)	Seconds	CT measured with kaolin activation and with heparinase I	103–153 s
Clot stiffness (CS)	hectoPascals (hPa)	CS measured with thromboplastin activation with hexadimethrine bromide	13.0–33.2 hPa
Fibrinogen contribution (FCS)	hPa	CS measured with thromboplastin activation and hexadimethrine bromide and abciximab	1.0–3.7 hPa
Platelet contribution (PCS)	hPa	Calculated from subtracting FCS from CS	11.9–29.8 hPa
Clot time ratio	Unitless	Calculated ratio of CT over CTH	N/A

**Table 3:** Statistical Analysis of Arterial Versus Venous Performance in the Quantra

Quantra Parameter	Venous (mean)	Arterial (mean)	p Value	Slope (95% CI)^†^	Y-Intercept (95% CI)^†^
CT	126.5 s	140.0 s	< 0.001*	1.11 (0.89–1.34)	−29.44 (−61.22 to 2.34)
CTH	129.9 s	142.7 s	< 0.001*	0.86 (0.63–1.08)	7.46 (−25.13 to 40.05)
CS	22.04 hPa	22.03 hPa	0.96	0.98 (0.93–1.03)	0.43 (−0.75 to 1.60)
FCS	3.44 hPa	3.4 hPa	0.53	1.04 (0.99–1.09)	−0.11 (−0.32 to 0.10)
PCS	19.03 hPa	18.95 hPa	0.76	0.96 (0.90–1.02)	0.82 (−0.44 to 2.08)
CTR	1.05	1.05	0.74	0.87 (0.77–0.97)	0.14 (0.03–0.24)

Abbreviations: CS, Clot Stiffness; CT, Clot Time; CTH, Heparinase Clot Time; CTR, Clot Time Ratio; FCS, Fibrinogen Contribution to clot stiffness; PCS,Platelet Contribution to clot stiffness.

* Statistically significant.

† Linear regression with venous as the y-variable and arterial as the x-variable.
